# ENG-releasing subdermal implants in postpartum teenagers – an open-label trial study protocol

**DOI:** 10.1186/s12978-020-00952-5

**Published:** 2020-06-23

**Authors:** M. M. Barbieri, C. R. T. Juliato, L. Bahamondes, F. G. Surita

**Affiliations:** Department of Obstetrics and Gynecology, School of Medical Science, University of Campinas, Av. Alexander Fleming, Campinas, SP 101 Brazil

**Keywords:** Subdermal implant, Contraception, Puerperium, Teenagers

## Abstract

**Background:**

Higher than expected adolescent pregnancy high rates continue globally, with repeated unplanned pregnancy (UP) in this age group is a public health problem. In Brazil, 16% of pregnancies occur in adolescents under 18 years old, with high maternal morbidity and mortality rates in this age group. Effective and safe contraception is required to reduce UP rates. The objective of our study is to evaluate acceptance of etonogestrel (ENG)-releasing subdermal contraceptive implant after childbirth, before discharge, as well as clinical performance up to one year after placement. Comparison between teenagers who opt for ENG-implant versus other contraceptive methods after childbirth will be also evaluated, specifically regarding UP, continuation and discontinuation rates and reasons, body composition, pelvic ultrasound characteristics and user satisfaction.

**Methods:**

A non-randomized open-label trial will be conducted with teenagers after childbirth and followed up to one year at the Women’s Hospital, University of Campinas (UNICAMP), Campinas, Brazil. The study group will consist of patients who accepted to use ENG-implant and placed before discharge. The comparison group will include adolescents who choose to use other contraceptive methods at the first postpartum visit (42 ± 3 days after childbirth). All women will follow-up at 40–60 days postpartum, as well as, at 6 and 12 months post-enrollment. Patient satisfaction, contraceptive effectiveness, reasons of discontinuation, continuation rate and body composition will be evaluated. Transvaginal ultrasound and electric bio impedance tests will be performed at all follow-up appointments. A 5% significance level was assumed, as well as, a sampling error (absolute) for 10% prevalence. The sample size was calculated at *n* = 100, obtaining an estimate of 50 to 70 adolescents who would accept the method offered, according to the prevalence and sample error assumed.

**Discussion:**

Long-acting reversible contraceptive (LARC) methods include subdermal implants and intrauterine contraceptives, are considered first line contraception for teenagers. Immediate postpartum use is a safe option, which significantly reduces rates of repeated UP and all the undesirable consequences inherent to this process.

**Trial registration:**

This study was approved by the Ethics and Research Commission of UNICAMP (CAAE: 92869018.5.0000.5404) and the Brazilian Registry of Clinical Trials (REBEC): http://www.ensaiosclinicos.gov.br/rg/RBR-4z7bc6, (number 2.901.752).

## Plain ENGLISH summary

Long-acting reversible contraceptives (LARC), which include the subdermal implant, are considered first line for teenagers. Immediate postpartum use is a safe option, which significantly reduces rates of repeated unplanned pregnancies, as well as all the undesirable consequences inherent to this process.

The aim of this protocol is to evaluate acceptability of the use of subdermal implant during the immediate postpartum period, its contraceptive efficacy, adverse effects and reasons for discontinuation over a one-year period. In addition, information regarding the relationship between implant use and body composition, ovarian ultrasound characteristics and user satisfaction were analyzed and compared to a group of teenagers using other contraceptive methods started during the postpartum period.

All patients aged up to 19 years, who give birth at the Woman’s Hospital will be invited to participate in the study prior to hospital discharge, and when the patient chooses to use the subdermal implant, it will be inserted immediately. The women that opt for other contraceptive methods, they will be offered at the return between 40 and 60 days postpartum. All women will follow-up at this first visit postpartum as well as at 6 and 12 months post-enrollment.

Our expectation is that almost 60% of the adolescents after childbearing at our hospital will choose the subdermal implant as contraceptive method due to the higher contraceptive efficacy when compared to non-LARC methods and few side-effects mainly abnormal uterine bleeding, with a high rate of continuation and satisfaction.

## Background

Pregnancy at adolescent age is higher than expected worldwide, particularly in low and middle-income countries (LMIC) and in Latin American and the Caribbean (LAC) countries is very high. Rates in LAC countries are the second highest, surpassed only by sub-Saharan African countries, at 66.5 in 1000 women. In Brazil, 16% of pregnant women are in this age group, with highest parity seen in those who became pregnant before the age of 20, representing the part of the population that contributes to continued high total fertility rates [1, 2].

Maternal mortality is one of the main causes of death in teenagers and young women up to 24 years of age. This, alongside maternal morbidity and near misses, are far more frequent when pregnancy occurs before the age of 15, when preeclampsia, postpartum hemorrhage and anemia are the main causes [3, 4]. Additionally, pregnant adolescent also present an increased risk of prematurity, small for gestational age (SGA) newborns and increased perinatal and childhood morbidity and mortality [5].

Multiple strategies have been adopted aiming to reduce these numbers, with educational interventions and promotion of effective and safe contraceptive methods for teenagers among the measures with the greatest positive impact [6]. UP among teenagers is a consequence to improper and/or lack of use of contraceptive methods [7]. LARC methods, which include subdermal implants and intrauterine contraceptives (IUCs) are the first line for this age group [8–10].

LARCs are the most effective reversible contraceptives, with a pregnancy rate of less than 1/100 women/year, as well as a high satisfaction, low discontinuation and high continuation rate in teenagers [8, 9]. Subdermal implants are one of the best method, with a failure rate of 0.4/100 women/year [11, 12]. In addition, immediate postpartum use is highly recommended, because it is a time when women are prone to prevent a new pregnancy and due to the fact that many women are unable to return to a health facility or do not have access to a LARC method [11, 13–16].

Despite LARC being recommended as the method of choice in these women, less than 5% of health professionals prescribe and insert them [8, 17]. Care and guidance on postpartum contraception during antenatal care is essential in planning and choosing the best method to prevention of new pregnancies in the short term [18].

Otherwise, placement of the ENG-implant is considered safe during breastfeeding and do not presents maternal, neonatal or postpartum risks [11, 13–16]. with no risk of expulsion as occurred with IUC [19].

Also, teenagers presented a high adherence rate when using LARC, with rates of 84 and 74% for the subdermal implant and IUD, respectively [20]. Abnormal uterine bleeding is cited as the main reason for withdrawal of the method [21].

However, a study with women who opted for insertion of the ENG- implant during the immediate postpartum period and followed-up over 3 years, showed that teenagers, who represented 28.2% of the sample, had high continuation rate (94.5%) [22].

The repetition of an UP among teenagers is associated with a low level of schooling, drug addiction and incorrect use of contraceptives among other causes [23]. In this population, the incidence of a new pregnancy within 2 years after the first one was 35%; however, with placement of any LARC at the immediate postpartum period, the rate decreased by 88.2% when compared to non-LARC methods [24].

The aim of our study is to evaluate acceptability, contraceptive efficacy, discontinuation rate by reasons and continuation rates among adolescents who either accept the ENG-implant versus non-acceptors at the immediate postpartum period and follow-up up to one year after placement. In addition, information regarding the relationship between implant use and body composition, pelvic ultrasound characteristics and user satisfaction will be analyzed in both groups.

## Methods/design

### Study type

A non-randomized and open-label trial.

### Setting

The study will be conducted at the Department of Obstetrics and Gynecology, University of Campinas Medical School, Campinas, Brazil. The facility is a tertiary referral public hospital wich offer treatment and it is referral for approximately 60 municipalities, covering a population of more than five million inhabitants.

### Inclusion and exclusion criteria

The inclusion criteria are all women aged up to 19 years old, who give birth at our hospital, and are invited to participate in the study prior to hospital discharge. Exclusion criteria are those who chose to not use any contraceptive method, or present an absolute contraindication to the use of the contraceptive methods offered according to the World Health Organization (WHO) eligibility criteria [25], as well as, those who are unable to attend follow-up appointments during the proposed study period.

### Intervention

Placement of ENG-implant (Implanon NXT, MSD, Oss, The Netherlands) at the immediate postpartum period (up to 48 h after childbearing). This method is not yet offered as routine in the hospital. After properly counselling (including effectiveness, characteristics, possible adverse effects, shelf-life use, and options to change the contraceptive method chosen after enrollment), the participants will be divided in two groups: 1) study group among those who chose ENG-implant and will receive it prior to hospital discharge and 2) non-acceptors of implant and acceptors of other contraceptive methods at the first follow-up between 40 and 60 days postpartum.

### Follow-up

All participant women will be schedule to return to postpartum visit up at 40–60 days, and again at 6 and 12 months post-enrollment. Patient satisfaction, contraceptive effectiveness, rates and reasons of discontinuation and continuation rate, as well as, body composition will be evaluated. Transvaginal ultrasound and electric bio impedance tests will be performed at all follow-up appointments. Non-implant acceptors will start follow-up at the 40–60 day after childbearing. Efficacy, characteristics, and contraindications will be based on the WHO criteria [25]. Women who do not attend the schedule follow-up visits and those who we were unable to contact after three telephone call will be consider lost-to-follow-up (LFU).

### Procedures and techniques

Guidance, insertion and removal of subdermal implants, as well as prescription of other contraceptive methods, electrical bioimpedance and outpatient follow-up will be performed by the researchers. A specialist in Gynecological and Obstetric Ultrasound will perform the ultrasound scans. Subdermal implant placement will follow the manufacture technique [26]. The implant will be inserted with the patient in the supine position, with the medial aspect of the upper arm exposed, the left arm when right-handed, or the right arm when left-handed, approximately 6 to 8 cm above the elbow crease, in the medial bicipital groove. Using an adequate aseptic technique, local anesthetic (2 ml of 1% lidocaine) will be applied, and the implant placed using its own application device in the subdermal connective tissue. After insertion, palpation will confirm its position. In situations where it is not possible to palpate the implant or should there be doubts regarding adequate positioning, an ultrasound scan will be performed for confirmation. Following insertion, occlusive dressings will be applied, and the User Card filled-out and given to the patient. Electric bioimpedance will be performed using bioelectrical impedance, with a single frequency unit (50 kHz), Quantum II Bia Analyzer® brand (Q-JL Systems, Michigan, USA). For this test, the patient will remain in the supine position with the electrodes placed at the following anatomical points: near the metacarpal-phalangeal and the metatarsal-phalangeal joints (current-emitting electrodes); between the distal prominences of the radius and ulna; and between the medial and lateral malleolus of the ankle (voltage sensing electrodes). This method can evaluate percentages of total body fat, fat free mass, dry lean mass, total body water and intracellular water. Ultrasound scans will be performed in the Diagnostic Imaging Sector of the Women’s Hospital using a Voluson 730 Expert device, via transvaginal, endocavitary probe, transvaginal preset, 6.5 MHz frequency, mode B, with focus, gain and depth adjusted. The average time of each US scan will be 15 min, with evaluation of the following parameters: uterine volume, endometrial appearance and thickness, ovarian volume and characteristics, and the presence of corpus luteum or other cysts. All images will be saved on the machine used, as well as an external hard drive.

### Sample size

**Fig. 1:**
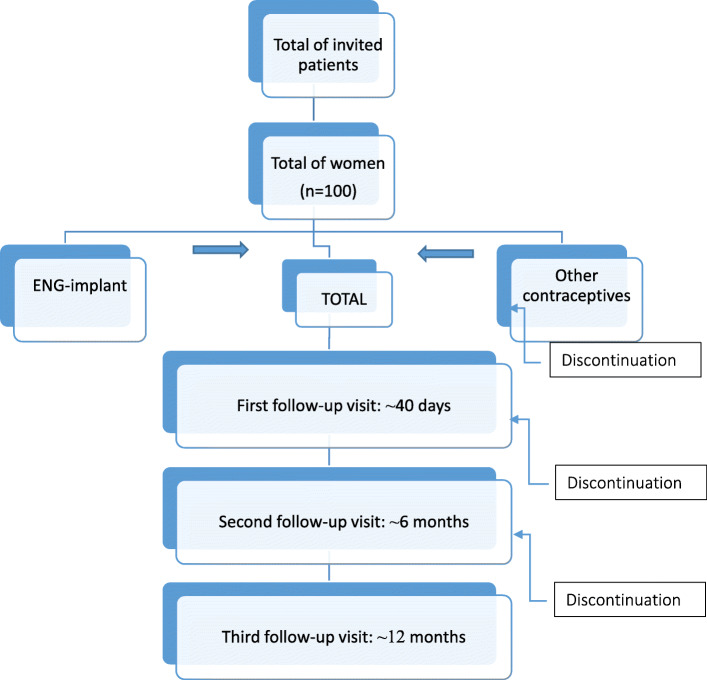
Expected Flowchart

The sample size was calculated based on a prevalence of acceptance of the ENG-implant, based on the satisfaction of teenagers users with the implant [27]. A significance level of 5% and a sampling error (absolute) for the prevalence of 10% were assumed (expected prevalence between 50 and 70%). The formula is based on a binomial distribution (satisfied or not satisfied) n = Z21-a/2 p(1-p) / e2, since e = sampling error of prevalence, p = estimated prevalence (based on literature), a = significance level and Z is the value of normal distribution. The sample size was calculated in *n* = 92,2 and rounded to 100 to contemplate casual lost in the sample. We expected obtaining an estimate of between 50 and 70 teenagers who will accept the method, according to the prevalence and sample error assumed. After collecting the data, the groups will be compared for the dependent variables and the power of the test will be calculated for user satisfaction, method effectiveness, side-effects, body composition, and ultrasound evaluation. The expected flowchart from the study is in Fig. 1.

### Statistical analysis

The data will be recorded in a data collection sheet prepared for this study. The encrypted data will be stored anonymously in a excel database, after manual review and double typing for descriptive statistical analysis (mean, standard deviation, absolute and relative frequency distribution). The association between independent and dependent variables will be performed using the X^2^test (for qualitative variables) or Fisher’s exact test (when 25% or more of the expected values ​​are less than 5) and through the Student’s *t* test (or the Mann-Whitney test if the data do not present normal distribution, as well as logarithmic, inverse and quadratic transformations of the same). Open questions will be categorized for application of statistical analyzes as well as qualitative variables. Additionally, we will estimated the couple year protection (CYP) which is an indicator of the number of pregnancies averted in each of the groups under evaluation. The level of significance adopted for the statistical tests will be 5%. For statistical analysis, the program Statistical Analysis System (SAS), version 9.4 will be used. Other procedures will be adopted for quality control. Each patient will have an identification number so as not to permit confusion of data, the completed forms will be reviewed and the data put on to the database by two different typists in order to avoid losses and typing errors. All material used for data collection will be stored by the researcher for five years, remaining confidential. This article has followed the SPIRIT guidelines for its elaboration [28].

## Discussion

Our expectation is that almost 60% of the adolescents after childbearing at our hospital will choose the ENG-implant as contraceptive method due to the higher contraceptive efficacy when compared to non-LARC methods and few side-effects mainly abnormal uterine bleeding. Breakthrown or unscheduled bleeding after ENG-implant placement is the main reason for early discontinuation; however, we expected that in this particular group of users, due to the physiological bleeding after childbearing, this side-effect could be avoided, they will be satisfy and continuation rate could be high than when placement of ENG-implant occurs at interval period.

In addition, it is unlikely that body composition will be affected with the use of the ENG-implant, because it was described that the weigh gain is almost one kg/year after implant placement, with most patients likely to present normal pelvic characteristics during ultrasound scans.

The large number of UP among Brazilian adolescents and young women is one of the main sexual and reproductive health public problem. One of the consequences is the high cost for the national health service of approximately US$ one billion per year and childbearing at adolescent age contribute significantly to this burden [29]. According to data from the Brazilian Ministry of Health, approximately 20% of all pregnant women are teenagers and, ~ 60% are UP. Therefore, we consider that our study could be a demonstration one to provide reliable data on one strategy about how to reduce these indices.

LARC methods, including the ENG-implant are the first option to reduce UP in teenagers [30]. However, there are few studies in the literature that assess its use in this specific population.

## Data Availability

Not applicable.

## Abbreviations

UP: Unplanned pregnancy
ENG-Implant: etonogestrel releasing subdermal contraceptive implant
UNICAMP: University of Campinas
LARC: Long-acting reversible contraceptive
REBEC: Brazilian Registry of Clinical Trials
LMIC: Low and middle-income countries
LAC: Latin American and the Caribbean
SGA: Small for gestational age
IUCs: Intrauterine contraceptives
WHO: World Health Organization
LFU: Lost-to-follow-up
CYP: Couple year protection
SAS: Statistical Analysis System
